# Pain Processing after Social Exclusion and Its Relation to Rejection Sensitivity in Borderline Personality Disorder

**DOI:** 10.1371/journal.pone.0133693

**Published:** 2015-08-04

**Authors:** Melanie Bungert, Georgia Koppe, Inga Niedtfeld, Sabine Vollstädt-Klein, Christian Schmahl, Stefanie Lis, Martin Bohus

**Affiliations:** 1 Department of Psychosomatic Medicine and Psychotherapy, Central Institute of Mental Health, Medical Faculty Mannheim, Heidelberg University, Mannheim, Germany; 2 Department of Addictive Behavior and Addiction Medicine, Central Institute of Mental Health, Medical Faculty Mannheim, Heidelberg University, Mannheim, Germany; Bellvitge Biomedical Research Institute-IDIBELL, SPAIN

## Abstract

**Objective:**

There is a general agreement that physical pain serves as an alarm signal for the prevention of and reaction to physical harm. It has recently been hypothesized that “social pain,” as induced by social rejection or abandonment, may rely on comparable, phylogenetically old brain structures. As plausible as this theory may sound, scientific evidence for this idea is sparse. This study therefore attempts to link both types of pain directly. We studied patients with borderline personality disorder (BPD) because BPD is characterized by opposing alterations in physical and social pain; hyposensitivity to physical pain is associated with hypersensitivity to social pain, as indicated by an enhanced rejection sensitivity.

**Method:**

Twenty unmedicated female BPD patients and 20 healthy participants (HC, matched for age and education) played a virtual ball-tossing game (cyberball), with the conditions for exclusion, inclusion, and a control condition with predefined game rules. Each cyberball block was followed by a temperature stimulus (with a subjective pain intensity of 60% in half the cases). The cerebral responses were measured by functional magnetic resonance imaging. The Adult Rejection Sensitivity Questionnaire was used to assess rejection sensitivity.

**Results:**

Higher temperature heat stimuli had to be applied to BPD patients relative to HCs to reach a comparable subjective experience of painfulness in both groups, which suggested a general hyposensitivity to pain in BPD patients. Social exclusion led to a subjectively reported hypersensitivity to physical pain in both groups that was accompanied by an enhanced activation in the anterior insula and the thalamus. In BPD, physical pain processing after exclusion was additionally linked to enhanced posterior insula activation. After inclusion, BPD patients showed reduced amygdala activation during pain in comparison with HC. In BPD patients, higher rejection sensitivity was associated with lower activation differences during pain processing following social exclusion and inclusion in the insula and in the amygdala.

**Discussion:**

Despite the similar behavioral effects in both groups, BPD patients differed from HC in their neural processing of physical pain depending on the preceding social situation. Rejection sensitivity further modulated the impact of social exclusion on neural pain processing in BPD, but not in healthy controls.

## Introduction

Physical pain serves as an alarm signal for the prevention of and reaction to physical harm. The experience of being ostracized causes different emotions, which are also often described as hurting and painful, and may similarly signal a potentially harmful situation. Physical pain is defined as the reaction to (possibly) body-damaging stimuli (International Association for Studying Pain, IASP), and social pain signals the (potential) occurrence of social exclusion that would threaten the fundamental human needs of belonging, self-esteem, control, and a meaningful existence [1, 2]. Correspondingly, Eisenberger et al. [3] suggested a common, phylogenetically old pathway for both response patterns.

In healthy participants, Eisenberger et al. [3] found that social exclusion led to an activation of the dorsal anterior cingulate cortex (dACC), the anterior insula, and the right ventrolateral prefrontal cortex (vlPFC). Because these brain regions are associated with the experience and regulation of physical pain distress, Eisenberger et al. [3] proposed that social pain is processed by the same functional brain network. A recent meta-analysis of neuronal correlates to social rejection confirmed the activation of the anterior insula during rejection. This analysis did not support an engagement of the dorsal ACC and vlPFC, but it found activation in more ventral parts of the ACC and the inferior frontal cortex [4]. Kross et al. [5] were the first to compare the experience of physical and social pain in healthy participants directly by using a within-subject design. In accordance with the meta-analysis by Cacioppo et al. [4], they found a shared activation in the anterior insula as well as an engagement of the dACC during the processing of both physical and social pain. They also reported overlapping activation in brain regions that are generally relevant for the processing of the sensory characteristics of pain, i.e., in the thalamus and the secondary somatosensory cortex.

Based on the idea of shared functional networks, several studies on healthy participants have focused on the modulating effect of social rejection on the experience of physical pain and have revealed heterogeneous findings. Eisenberger et al. [6] reported that enhanced experiences of exclusion go along with increasing pain sensitivity, whereas DeWall & Baumeister [7] reported reduced pain sensitivity after the experimental induction of exclusion. After reviewing the literature and the applied paradigms to induce experiences of exclusion, Bernstein & Claypool [8] proposed a severity hypothesis in which the intensity of social rejection modulates pain processing. An analog to physical pain, in which mild injuries increase the pain experience but severe injuries lead to analgesia, the investigators propose that mild forms of social exclusion enhance sensitivity to physical pain, whereas more severe forms reduce sensitivity to physical pain. In a subsequent experimental study, the authors were able to support that hypothesis [8]. Because this finding suggests that the severity of the exclusion experience is a relevant factor in the interplay between social and physical pain, individual differences in the sensitivity to rejection and thus the strength of the effects of an experimentally induced exclusion seem important. Downey & Feldman [9] introduced the concept of rejection sensitivity as follows: a cognitive-affective disposition of individuals that differentiates between subjects in the severity with which they expect, perceive, and react to social rejection.

However, when investigating the influence of social rejection on physical pain, only a single study so far has accounted for the individual level of rejection sensitivity [10]. In this study, only participants with high levels of rejection sensitivity showed reduced sensitivity to pain, i.e., enhanced pain thresholds and reduced discomfort ratings after the experience of social exclusion compared with inclusion. In contrast, participants with low levels of rejection sensitivity were more sensitive to pain after social exclusion in the form of a reduced pain tolerance [10]. In addition, evidence from a genetic study also emphasizes the link between physical pain sensitivity and rejection sensitivity [11], in that the A118G polymorphism of the mu-opioid receptor gene, which has previously been linked to enhanced pain sensitivity, was also associated with greater rejection sensitivity. Taken together, these findings underline the importance of rejection sensitivity in the interplay between physical and social pain.

With regards to the hypothesized close connection between physical and social pain, borderline personality disorder (BPD) constitutes a pathological state of particular interest; the pain systems seem to be dissociated in this disorder, i.e., a hyposensitivity to physical pain [12] goes along with a hypersensitivity to social pain [13, 14].

Several studies have revealed reduced physical pain sensitivity in BPD, i.e., enhanced pain thresholds and tolerance as well as reduced pain intensity ratings compared with healthy controls [12, 15–19]. The observed alterations in subjective experience go along with alterations on the neuronal level, which have been found in cerebral regions that were linked to the affective and cognitive-motivational component of pain processing. Schmahl et al. [12] observed a reduced activation in the ventral ACC (vACC) and the right amygdala during pain processing, and both were associated with the affective pain component in BPD patients compared with healthy control subjects (HC) [20]. The authors also observed a reduced activation in the posterior parietal cortex (PPC) and an increased activation in the left dorsolateral prefrontal cortex (dlPFC), which are both relevant for the cognitive-evaluative component of pain [21, 22]. However, several studies suggest that the sensory-discriminative component of pain processing, unlike the affective and cognitive components, is not affected in BPD [18, 23, 24]. Only one study has reported reduced discriminative abilities in a sub-group of BPD patients, with analgesia reported during self-injurious behavior (SIB) compared with BPD patients who were experiencing pain during SIB and with non-clinical control subjects [25]. In summary, these findings suggest that hyposensitivity to pain in BPD is primarily linked to alterations in the affective and cognitive-motivational pain components.

In contrast to their hyposensitivity to physical pain, BPD patients are characterized by a hypersensitivity to social pain [13, 14]. This trend is partly reflected by enhanced rejection sensitivity, which is a personality trait consisting of concerns regarding social rejection and low expectancy towards social acceptance [9]. In the rejection sensitivity questionnaire (RSQ; [9]), BPD patients report a greater tendency to expect and perceive rejection in social situations and to be more concerned about these experiences compared with healthy individuals and patients with mood or anxiety disorders [26, 27]. Because of this enhanced expectation of exclusion, objective situations of inclusion may also be challenging for BPD patients because these situations may be highly unexpected. Experimental studies that employ the cyberball paradigm [28] support this idea and point to a biased perception of social interactions. In the cyberball paradigm [28], participants take part in an online ball-tossing game with virtual partners. The participants are told that they are playing with other study participants, but in fact, the co-players are pre-programmed by the experimenter to either include or exclude the subject. Compared with the HC, BPD patients experience stronger feelings of exclusion during the cyberball inclusion condition as well as under a control condition, during which the interaction follows predefined rules and is not determined by the motivation of the co-players [14, 26]. The first data also indicate alterations in cerebral processing during the cyberball paradigm in BPD in comparison with healthy controls. BPD patients showed an unspecific hyperactivation in the dACC and parts of the dorsal medial prefrontal cortex (dmPFC) compared with the HC [26, 29]. In healthy subjects, the engagement of the insula, the more anterior part of the dmPFC, the precuneus, and the dlPFC were modulated by the nature of the preceding social interaction, and no comparable effect was observed in BPD patients [26, 29].

Studies combining both types of pain as well as studies linking physical pain to rejection sensitivity are still missing in BPD studies. The aim of the present study was to investigate the influence of social pain on physical pain in BPD by measuring physical and social pain in the same individuals as well as exploring the influence of rejection sensitivity within this relation.

We applied the cyberball paradigm in combination with the application of painful heat stimuli during a functional Magnetic Resonance Imaging (fMRI) block design. Findings regarding social pain have been reported in Domsalla et al. [26]. Their analyses revealed that the subjective experience of exclusion was significantly higher during the exclusion compared with the inclusion or the control condition with predefined rules in both BPD patients and HC. As expected, the experience of inclusion was significantly higher during the inclusion and the control condition compared with the exclusion condition. This finding suggests that the experimental manipulations successfully induced the experience of social exclusion and inclusion. In addition, BPD patients differed from HCs during the inclusion and the control condition. Under these conditions, BPD patients reported stronger feelings of exclusion and lower ratings of inclusion compared with HC. By contrast, BPD patients did not differ significantly from HC in the exclusion or inclusion experiences during the social exclusion condition [26]. Taken together, these findings provide evidence for a biased perception of social belonging in positive or ‘neutral’ social situations [26].

The present study focuses on whether a preceding social interaction situation influences the subjective experience and the cerebral processing of physical pain, and whether rejection sensitivity modulates the difference between pain processing after social exclusion and inclusion in BPD. In this context, we tried to answer the following four research questions:

1. Can we replicate the previous findings of reduced pain sensitivity in BPD patients?

Here, we hypothesized that a temperature stimulus with a higher objective temperature is necessary to induce physical pain in BPD patients. According to previous findings [12], we expect to find differences in brain activation during pain processing that are independent of the preceding social interaction. We hypothesized that brain areas with reduced activation would be linked to the affective pain component, such as the vACC and the amygdala, and with enhanced activation, they would be located in areas related to the cognitive-motivational pain component, such as the dlPFC during pain in BPD patients compared with HC.

1. Does social exclusion lead to a hypersensitivity to pain when measured in comparison with social inclusion or a control condition with predefined rules?

Because HC and BPD patients experience stronger exclusion during the exclusion condition compared with the inclusion and control conditions [26], and because cyberball is evaluated as a mild form of rejection [8], we hypothesized that HC and BPD patients would experience higher pain ratings after social exclusion. These higher pain ratings should go along with enhanced activation during pain after exclusion compared with the inclusion and the control condition in pain-related brain regions.

1. Do BPD patients differ from HC in the way in which they experience physical pain after social exclusion, social inclusion, and a control condition with predefined rules on a subjective level and on the neuronal level?

We expected to identify group differences in the experience and neuronal processing of physical pain in BPD after the experience of social exclusion. Because of the enhanced experience of exclusion during the inclusion and the control condition in the patient group (see [26]), we were also interested in finding whether the effects of these interaction situations on subsequent pain processing are altered in BPD.

1. Does rejection sensitivity influence the experience and neuronal processing of physical pain after social exclusion and inclusion?

Because rejection sensitivity influences the way people experience exclusion, and because pain experiences seem to be altered after exclusion in a way that is dependent on the level of rejection sensitivity [10], we expected to find associations between the RS level and the experience as well as neuronal processing of physical pain after applying the different cyberball conditions.

## Methods and Material

### Subjects

Twenty female patients who met the DSM-IV-criteria for BPD (aged 28.7 years, SD = 7.8) and twenty healthy female participants (HC) without any lifetime or current psychiatric diagnosis (aged 29.2 years, SD = 7.5) participated in our study (see also [26]). We recruited the patients from our department database, and the HCs were contacted by newspaper advertisement. All patients were outpatients at the time of the investigation. The subject groups were matched for age and educational level. We included participants between 18 and 45 years. The general exclusion criteria were a lifetime history of psychotic disorder, current major depression, present substance abuse or addiction, pregnancy, organic brain disease, a history of skull or brain damage, severe neurological illnesses, current use of psychotropic medication, metal in the body, left-handedness, and claustrophobia. The International Personality Disorder Examination (IPDE, [30]; interrater reliability: κ = .69) was conducted by trained clinical psychologists to assess the BPD diagnosis. An N = 10 out of N = 20 fulfilled the IPDE criterion of self-harm behavior in our BPD sample. Co-morbid DSM-IV axis-I disorders were assessed by using the German version of the Structured Interview for DSM-IV (SCID-I, [31]; interrater reliability: κ = .77). We applied several self-rating-questionnaires to assess the borderline symptom severity (Borderline-Symptom-List, BSL; [32]), general psychopathology (Brief Symptom Inventory (BSI; [33]), and depressive symptoms (BDI; [34]). Additionally, we assessed rejection sensitivity (Adult Rejection Sensitivity Questionnaire, A-RSQ; [9, 35]) as a relevant personality trait. Table 1 contains the relevant subject characteristics. This study followed the Declaration of Helsinki and was approved by the Research Ethics Board of the University of Heidelberg. Each participant provided written informed consent prior to study participation.

**Table 1:** Demographic and clinical variables in patients with borderline personality disorder (BPD) and in healthy controls (HC) with results of the t-tests (independent, two-tailed).

	BPD-patients			HC			BPD vs. HC (independent t-test)	
	n	Mean	SD	n	Mean	SD	T	p
Age	20	29.2	7.5	20	28.7	7.8	-0.2	.800
Years of education	20	12.1	1.5	20	12.1	1.5	0.0	1.000
RSQ	20	14.1	5.0	20	5.5	2.7	-6.8	<.001
BSL	20	1.6	0.6	19	0.2	0.2	-9.7	<.001
BDI	20	18.1	9.9	20	2.3	2.9	-6.8	<.001
BSI	20	1.3	0.5	19	0.2	0.2	-8.4	<.001
Comorbidity	current	lifetime						
Major depression	0	18						
Bipolar-II	0	2						
PTSD	5	5						
Panic Disorder	1	5						
Social Phobia	5	7						
Specific Phobia	5	6						
OCD	1	4						
Bulimia	1	4						
Anorexia	0	4						
Substance Abuse/ Dependence	0	7						

PTSD, Posttraumatic Stress Disorder; OCD, Obsessive-Compulsive Disorder

### Functional and Structural MRI Acquisition

A Siemens TRIO-3T MRI scanner (Siemens Medical Systems, Erlangen, Germany) was used to obtain brain images. High resolution anatomical scans using a T1-weighted 3-D magnetization-prepared-rapid-acquisition-gradient-echo (1x1x1 mm³ voxel size) were acquired for each participant. The blood oxygen level-dependent (BOLD) signal was measured by using T2-weighted gradient echo planar imaging (EPI) with the following protocol parameters: field of view = 192x192 mm^2^, voxel size = 3x3x3 mm³, echo time = 30 ms, TR = 2000 ms, number of slices = 36, slice thickness = 3 mm, and matrix = 64x64. To minimize the T1 effects, the first five scans were discarded.

### Experimental design and tasks

Within an fMRI block design, the subjects played a total of 18 blocks of a virtual ball tossing game (cyberball, [3, 28, 36]) with two virtual partners (see [26]). Each ball tossing block was followed by the administration of either a painful (subjective pain intensity = 60%) or non-painful temperature stimulus. The subjects were then asked to assess the pain intensity of the temperature stimulus and the subjective experience of being excluded and included during ball tossing as well as their level of inner tension and dissociative symptoms. The cyberball and temperature stimuli conditions were presented in a pseudorandom order. Presentation (NeuroBehavioral Systems, Inc; Berkeley, CA, USA) was used as experimental control software. A LumiTouch fMRI Optical Response Keypad (Photon Control Inc.; Burnaby, BC, Canada) was used as a patient response system.

### Induction of social exclusion with the cyberball paradigm

All subjects played the ball-tossing game under three conditions. During social exclusion, participants received the ball only once at the beginning of each block and were excluded during the rest of the ball tossing block. During social inclusion, all participants received an equal number of ball tosses. To assess the influence of ascribed intentionality during the inclusion condition, we added a control condition with predefined game rules during which the participants also received an equal number of ball tosses. Under this condition, the sequence of ball tosses could not be freely chosen. Participants were instructed to toss the ball only to their right, or only to their left partner. Thus, receiving or not receiving a boll toss under this condition could, at least objectively, not be ascribed to the actual intention of the other virtual partner. Each subject underwent 6 blocks per condition (mean duration 30 s), which resulted in a total of 18 blocks of cyberball. To increase the ecological validity, photos of the alleged co-players were presented throughout the ball tossing game (see [28]).

### Pain application

Temperature stimuli (duration 30 s) were delivered to the inner side of the left forearm by using a thermode (3x3 cm^2^) controlled by a quantitative sensory tester (TSA-II; Medoc Advanced Medical Systems, Ramat Yishai, Israel). For pain stimuli, the temperature was chosen individually to correspond to 60% pain intensity on a visual analog scale (VAS). A neutral temperature stimulus (32°C) served as the control condition. Pain and warmth stimuli were presented equally often after each cyberball condition in a pseudo-randomized order.

A pain intensity of 60% was determined by following a standard procedure prior to the experiment; the stimulation temperature started at 37°C in time blocks of 30 seconds, oscillating +/- 1°C, and was increased in 1°C steps (above 41°C, in 0.5°C steps) until a subjective pain intensity of 60% was reached.

### Subjective Ratings during the experiment

To assess the experience of social exclusion after each temperature stimulation, the subjects had to rate their feelings of exclusion and inclusion and the percentage of received ball tosses as well as the extent of inner tension, dissociative symptoms (DSS-4; [37]), and the painfulness of the temperature stimulus. The ratings were assessed on an 11-point-visual scale ranging from “not at all” to “very strong” (0% to 100% for number of ball-tosses, respectively). The results for the experiences of social exclusion and inclusion, dissociation, and inner tension are reported in Domsalla et al. [26].

### Data analysis

#### Behavioral data

Differences in the subjective pain ratings of the painful stimuli after the cyberball conditions were tested by using 2x3-repeated-measure analyses of variance with a ‘group’ between-subject factor (HC and BPD) and a ‘cyberball condition’ within-subject factor (exclusion, inclusion, and control). To analyze the interaction effects further, post-hoc tests with Bonferroni correction for multiple comparisons were applied. Group differences in personality traits (rejection sensitivity and self-esteem) as well as differences in the pain temperatures corresponding to 60% pain intensity were calculated by using two-sample t-tests. Moderator analyses were used to test associations between subjective ratings, personality traits, and brain activations by considering the group as a potential modulating factor. The analyses were conducted by using the PROCESS macro written by Andrew F. Hayes [38], which can be implemented in SPSS, and it is based on an ordinary least squares regression. SPSS (SPSS Statistics 20; IBM Corporation, Armonk, New York, USA) was used as statistical software.

#### fMRI data

Functional imaging data were analyzed by using the standard procedures that were implemented in the statistical parametric mapping software package (SPM8; Welcome Department of Cognitive Neurology, London, UK). The preprocessing of the EPI time series was conducted by following custom practice (with a slice time correction and spatial realignment to correct for head motion and co-registration onto participants’ T1-scan; normalization to the standard brain of the Montral Neurological Institute (MNI) space; resampling to 3x3x3 mm³ voxels; and smoothing with a Gaussian kernel with a full-width at half the maximum of 9 mm). For regressors to model the first level analysis, we used the following factors: 3 regressors for the cyberball blocks exclusion, inclusion, and control; 3 regressors for the pain blocks after the different cyberball conditions; 3 regressors for the non-painful temperature blocks after the different cyberball conditions; and 1 regressor modeling key press. The relevant first-level-contrast images were entered into a second level analysis full-factorial-model. The effects of social exclusion on pain processing were analyzed in a 2x3x2-factorial model (‘group’ x ‘cyberball condition’ x ‘temperature’), which included six contrasts (two options: ‘pain’ and ‘warmth’ after 3 conditions: ‘exclusion’, ‘inclusion’ and ‘control’) for each group. The six realignment parameters were entered as additional regressors into the model. Region of Interest (ROI) analyses using small volume correction were conducted to investigate regions that were previously associated with the overlap of social and physical pain [3, 5], i.e., the insula, anterior cingulate cortex, thalamus, and ventrolateral prefrontal cortex. The amygdala and dorsolateral prefrontal cortex were also added as ROIs because both regions have been related to pain processing [39, 40] and are assumed to be linked to altered pain processing in BPD [12].

Masks were created by using anatomical templates as provided by the WFU PickAtlas v3.0 [41, 42]. Analyses were conducted by applying a lowered significance level of p = .01, but only clusters that survived a family-wise error (FWE) correction for multiple comparisons on the voxel level within the ROIs when using a small volume correction (p_SVC-FWE_<.05) are presented. Peak voxels are reported as MNI coordinates [x y z]. Because of the exploratory character of the study with respect to the influence of social pain on physical pain in BPD, no experiment-wise adjustment of the alpha level for the different ROIs was performed to avoid the inflation of type II error [43].

For the ‘temperature’ main effect, two directed t-contrasts were calculated (painful stimuli > non-painful stimuli, non-painful stimuli > painful stimuli). For the interaction effect ‘group’ x ‘temperature’ directed t-contrasts were calculated to investigate activation differences during painful as opposed to non-painful stimuli between the BPD and the HC group. To analyze group differences further between pain processing after the different social encounters, two directed t-contrasts were built to compare painful and non-painful stimuli between both groups separately for each cyberball condition (exclusion, inclusion, and the control condition). To specify the observed group effects further, additional interaction contrasts were built by comparing group differences in pain processing after social exclusion with group differences in pain processing after social inclusion and after the control condition in all the ROIs with significant group differences after one of the cyberball conditions. The enhanced ratings for exclusion during the inclusion and the control condition in BPD might confound the results of the group x temperature interaction after social inclusion and the control condition. To take this consideration into account, we conducted additional repeated measurement ANOVAs with the exclusion ratings during social inclusion and the control condition as a covariate.

Additionally, regression analyses were performed to investigate the influence of rejection sensitivity on the difference between brain activation during pain after social exclusion compared with pain after social inclusion separately for both groups.

## Results

### Behavioral Data

#### Temperature corresponding to 60% pain intensity

The temperature corresponding to a subjective pain intensity of 60% was significantly higher in BPD patients than in healthy subjects (BPD: mean = 44.3°C, sd = 2.0; HC: mean = 42.5°C, sd = 2.0; and t = -2.8, p<0.01). Moderator analyses did not reveal significant associations between this pain temperature and rejection sensitivity as measured by the RSQ, either for BPD patients or for HC (overall regression model: F[3,36] = 3.13, p = .038; p of all beta weights for group, RSQ and the interaction of group and RSQ >.05).

#### Pain ratings following cyberball conditions

Subjective pain ratings were modulated by the preceding interaction situation (see Table 2). Post-hoc tests showed that the pain ratings were higher after social exclusion than after social inclusion and the control condition. No differences between groups were observed, either in general or depending on the preceding ball tossing condition. No statistically significant relation was found between the pain experienced after the three cyberball conditions and the RSQ, either in the BPD group or in the HC group (overall regression model: F[3,36] = 0.60, p = .621; and p of all beta weights for group, RSQ and the interaction of group x RSQ of the moderator analysis >.05).

**Table 2:** Subjective pain ratings following the different cyberball conditions together with results of the 2x3-rm-ANOVA.

	BPD	HC	Statistics (2x3 rmAnova)		
Pain rating after	Mean (sd)	Mean (sd)	Group	Condition	Interaction
Exclusion	5.3 (1.9)	5.3 (1.1)	F(1,38) = 0.2	F(2,76) = 8.8	F(2,76) = 0.3
Inclusion	4.6 (1.7)	4.9 (1.2)	p = 0.698	p<0.001	p = 0.720
Control	4.4 (1.7)	4.6 (1.4)	η^2^_partial_ = .004	η^2^_partial_ = .188	η^2^_partial_ = .009

### fMRI Data

#### Main effect of temperature

ROI analyses revealed a significant main effect of the temperature on pain-relevant brain areas. Those areas showed higher activation during painful stimuli compared with non-painful stimuli: in the bilateral dACC, the insula and the amygdala as well as in the right thalamus and the right dlPFC (for further details see supporting information, S1 and S2 Tables). No brain regions were found to have higher activation during non-painful stimuli compared with painful stimuli.

#### Interaction effect condition x temperature

**Fig 1:**
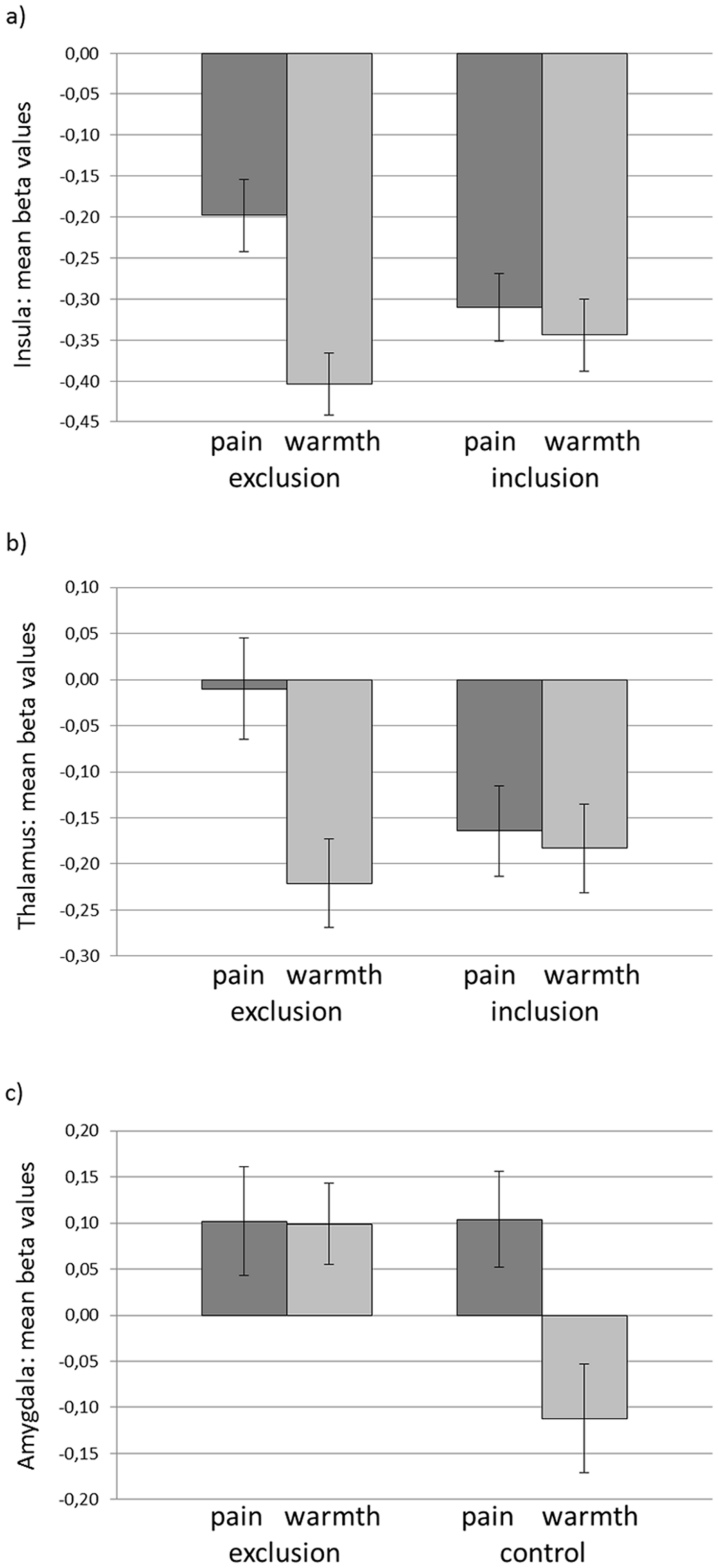
Mean beta values of the comparison between pain (vs. warmth) after social exclusion compared to inclusion a) in left insula, b) in right thalamus and c) compared to the control condition in the right amygdala independent of group.

We analyzed the interaction effect of ‘condition’ x ‘temperature’ by using directed t-contrasts to investigate the influence of social exclusion on pain processing independent of the group. Activation during pain (vs. warmth) was higher in the left anterior insula ([-27 23 1], p_SVC-FWE_ = .030) and the right thalamus ([3 -16 1], p_SVC-FWE_ = .050) (see Fig 1a and 1b) after social exclusion than after inclusion. A comparison of the pain (vs. warmth) experienced after social exclusion with the pain (vs. warmth) experienced after the control condition revealed a significant effect in the right amygdala ([27 2 -23], p_SVC-FWE_ = .037). This effect can be explained by reduced amygdala activation during warmth after the control condition (see Fig 1c).

No relations in the differential activation between painful and non-painful stimuli with the RSQ could be observed in the moderation analyses (overall regression model: F[3,36] = 1.03, p = .391 for the insula; F[3,36] = .69, p = .562 for the thalamus; F[3,36] = 2.28, p = .095 for the amygdala; and p of all beta weights for group, RSQ and the interaction of group and RSQ >.05).

#### Interaction effect group x temperature

ROI analyses revealed higher activation in the right posterior insula ([33 -31 19], p_SVC-FWE_ = .020), and there was a statistical trend of lower activation in the right amygdala ([33 2 -26], p_SVC-FWE_ = .065) during painful compared with non-painful stimuli, which was higher in the BPD group compared with the HC. No differences were observed in the ACC, thalamus, vlPFC, or dlPFC (all p_SVC-FWE_>.1).

Moderation analysis revealed a significant impact of the group (β = -3.34, p = .020) and the interaction group x temperature (β = .08, p = .019) on insula activation (overall regression model: F[3,36] = 4.64, p = .008). Additional conditional effect analyses showed a significant relation between the temperature of the applied pain stimuli and insula activation only in the BPD group (BPD: β = .08, p = .002; and HC: β = .002, p = .918).

No relations between the amygdala activation and the temperature of the applied stimuli was observed in the moderation analysis (overall regression model: F[3,36] = 3.31, p = .031; and p of all beta weights for group, temperature and the interaction of group x temperature of the moderator analysis >.05). The activation differences between painful and non-painful stimuli in the insula (overall regression model for pain ratings/RSQ: F[3,36] = 1.92, p = .143/ F[3,36] = .69, and p = .563) and the amygdala (overall regression model for pain ratings/RSQ: F[3,36] = 2.79, p = .054/ F[3,36] = 2.86, and p = .050) were not linked to mean pain ratings or the RSQ, and no moderation effect of the group was observed (p of all beta weights for group, pain ratings/RSQ and the interaction of group x RSQ/group x pain ratings of the moderator analysis >.05).

#### Interaction effect group x temperature following social exclusion

To test for the differential effects of the preceding cyberball conditions, we analyzed the interaction effects of the ‘group’ and ‘temperature’ factors separately for social exclusion, social inclusion, and the control ball-tossing condition.

**Fig 2:**
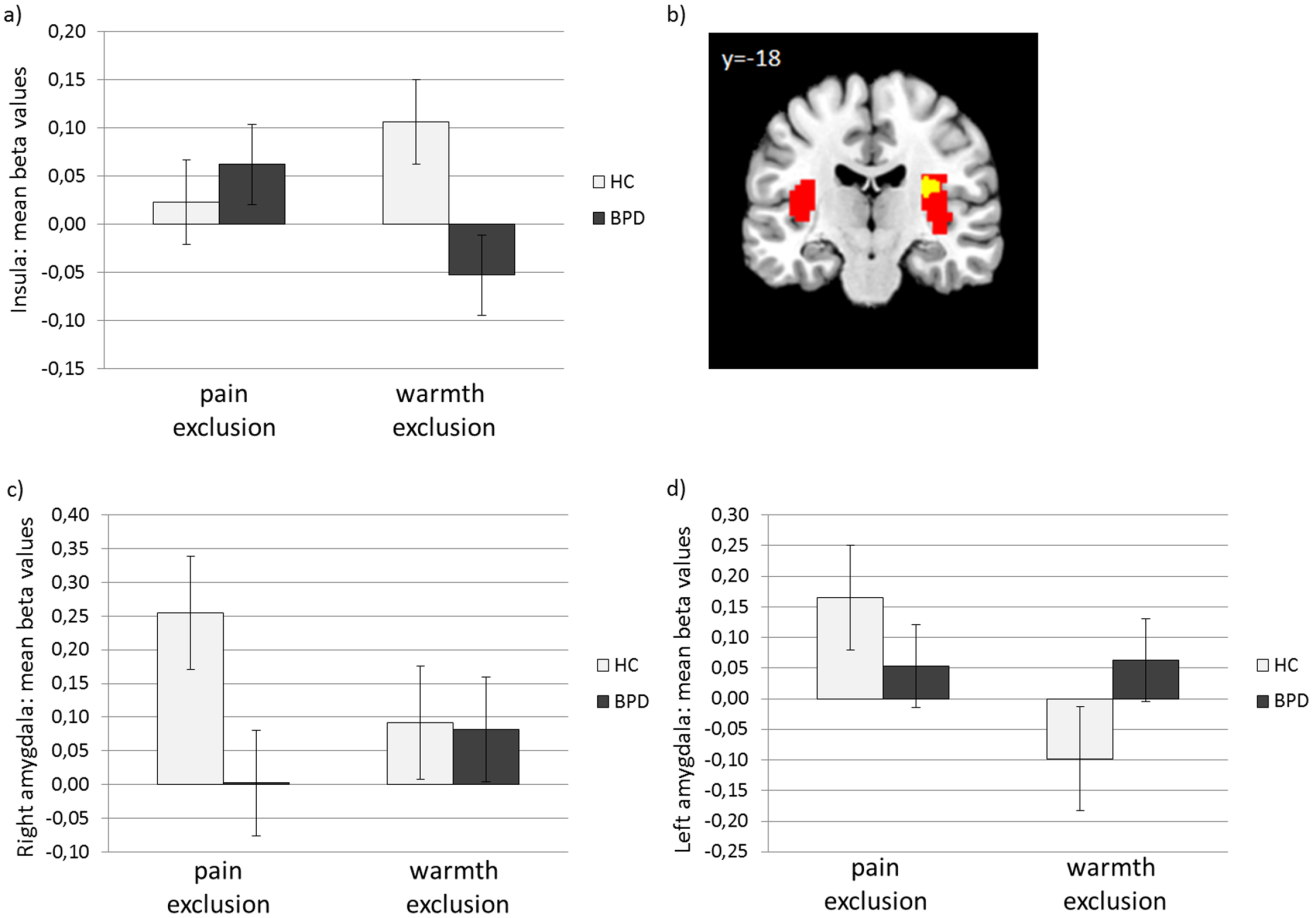
Insula and amygdala mean activation during painful compared to non-painful stimuli following social exclusion. a) mean beta values of peak voxel activation for HC and BPD in the insula, b) insula activation during pain after social exclusion independent of group (red), and the overlap of the general pain activation and the insula activation enhanced in BPD (yellow) (threshold for display: p < 0.005 uncorrected); mean beta values of peak voxel activation within c) the right amygdala, d) the left amygdala.

Activation during physical pain after social exclusion was higher in the right insula and showed a trend towards a lower activation in the amygdala for BPD patients compared with HC (see Fig 2a, 2b, 2c, and 2e; Insula: [30 -28 19], p_SVC-FWE_ = .035; amygdala: right [21 -7 -14], p = .074; and left: [-33 2 -26], p_SVC-FWE_ = .084).

For the insula and amygdala activation in the right hemisphere, different modulating variables were observed in both groups through moderation analyses.

First, the association between the temperature and insula activation depending on the group was tested in a multiple regression model (F[3,36] = 3.12, p = .038). These analyses revealed a significant impact of the group (β = -2.75, p = .025) and an interaction of the group x temperature (β = .06, p = .024) on insula activation. Additional conditional effect analyses showed a significant relation between the temperature of the applied pain stimuli and the insula activation only in the BPD group (BPD: β = .07, p = .001; and HC: β = .008, p = .672). In the next steps, we conducted further moderation analyses to test for possible relations between insula activation and pain ratings and the RSQ for both groups, which could not be observed (overall regression model for pain ratings: F[3,36] = 1.69, p = .187; and overall regression model for RSQ: F[3,36] = 1.32, p = .282).

Equivalent moderation analyses were conducted with right and left amygdala activation as a dependent variable. The analyses did not support a relation between the temperature of the applied stimuli and amygdala activation for both groups (overall regression model for right amygdala: F[3,36] = 0.87, p = .465; and overall regression model for left amygdala: F[3,36] = 1.55, p = .219). However, the moderation analyses revealed a relation between the increased intensity ratings of the experienced painfulness of the stimuli, the group and the interaction of group x pain ratings on right amygdala activation (overall regression model: F[3,36] = 2.97, p = .045; group: β = .93, p = .047; pain rating: β = .39, p = .014; and interaction group x pain rating: β = -.21, p = .017). Further conditional effect analyses showed a significant relation between pain ratings and right amygdala activation only in the HC group (HC: β = .18, p = .017; and BPD: β = -.03, p = .494). These relations were not observed in a comparable analysis of the left amygdala (overall regression model: F[3,36] = 2.30, p = .094; and p of all beta weights for group, pain ratings and the interaction of group x pain ratings of the moderator analysis >.05).

The overall regression models of the moderation analyses linking the RSQ with amygdala activation revealed a statistical trend for an association between those variables (F[3,36] = 2.73, p = .058 for right amygdala and F[3,36] = 3.00, p = .043 for left amygdala). However, in both models, neither the group nor the RSQ nor the interaction group x RSQ reached significance as a predictor in the model (all p>.05).

The described significant group effect for the insula could not be confirmed by using interaction contrasts for comparing group differences in pain processing after social exclusion in a direct way against group differences in pain processing after applying the control condition.

#### Interaction effect group x temperature following social inclusion

**Fig 3:**
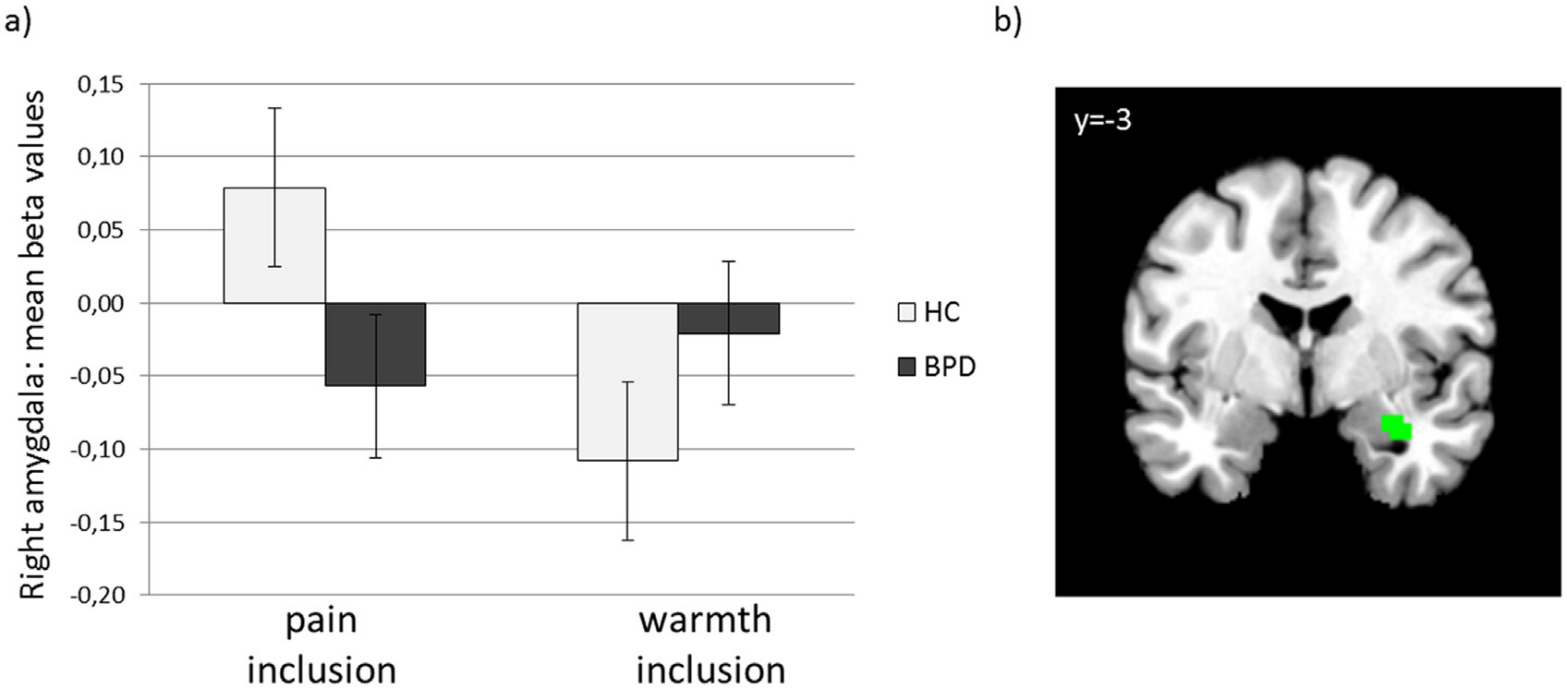
Mean amygdala activation during painful compared to non-painful stimuli following social inclusion. a) mean beta values of peak voxel activation for HC and BPD, b) visualization of reduced amygdala activation in BPD compared to HC during painful compared to non-painful stimuli following social inclusion (Threshold for display: p < 0.005 uncorrected).

ROI analyses revealed lower activation during painful stimuli than during non-painful stimuli in BPD patients compared with the HCs in the right amygdala ([33 -1 -23], p_SVC-FWE_ = .014), see Fig 3a and 3b). The additionally conducted repeated measurement ANOVA with the exclusion ratings during social inclusion as covariate did not reveal a significant effect for the exclusion ratings (F = .476, p = .495), and there is still a significant group x temperature interaction during pain processing after social inclusion in the activation of the right amygdala (F = 12.587, p = .001). Amygdala activation was not associated with RSQ, objective pain temperature or pain ratings after inclusion, either in the BPD patients or in the HC group (all p>.05 for the overall regression models of the moderation analyses).

The described group effects could be confirmed for the right amygdala (slightly different coordinate 24–1–14, p_SVC-FWE_ = .032) by comparing group differences in pain processing after social inclusion directly with group differences in pain processing after applying the control condition.

#### Interaction effect group x temperature following the control condition

In the ROI analyses, no clusters reached the significance level of p_SVC-FWE_<.05 for the group comparisons between activations during painful compared with non-painful stimuli following the application of the control condition.

#### Relations between rejection sensitivity and pain processing after social exclusion

To analyze the relations further between rejection sensitivity and pain processing, we calculated a regression analysis with RSQ scores and brain activation in pain-relevant ROIs during the differential processing of painful stimuli after social exclusion compared with social inclusion separately for each group.

**Fig 4:**
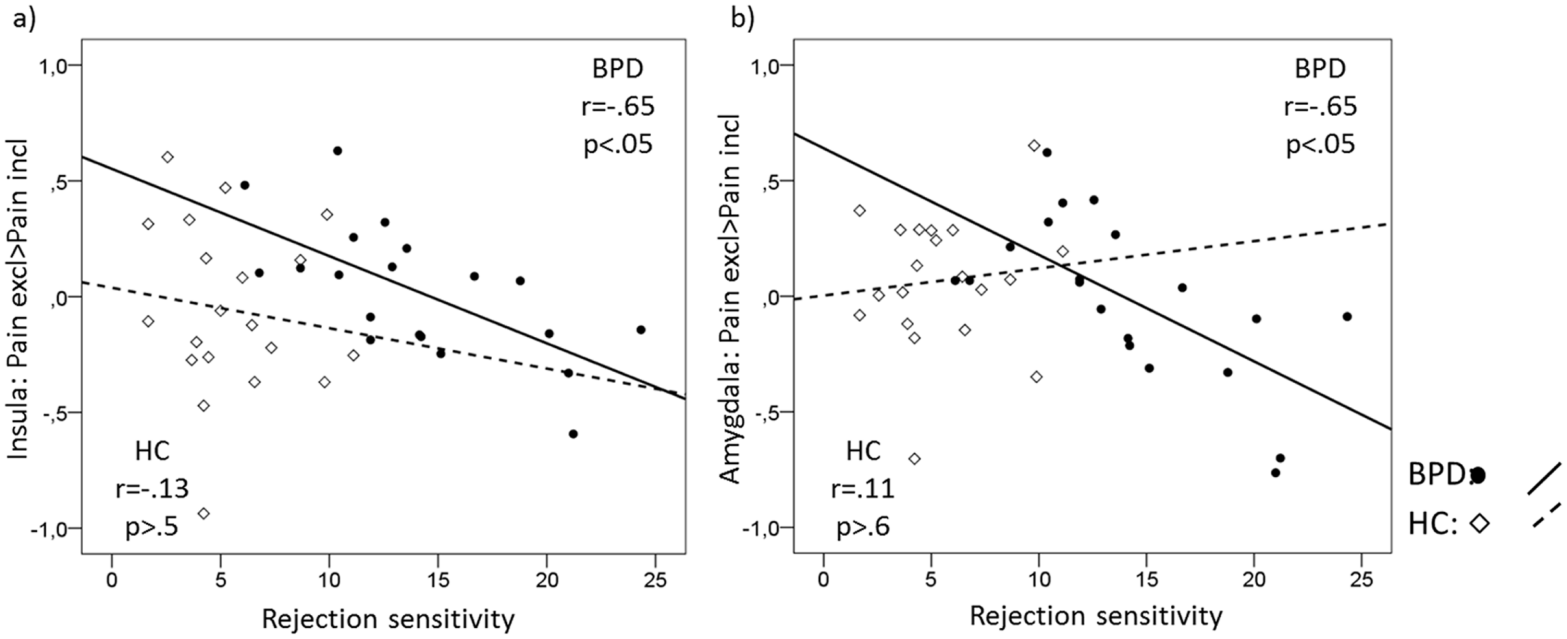
Co-variation between rejection sensitivity and a) mean insula activation, and b) mean amygdala activation during the processing of painful stimuli following social exclusion compared to social inclusion.

In the BPD group, higher rejection sensitivity was significantly associated with lower activation differences between pain after social exclusion and pain after social inclusion in the right insula ([42 -1 -5], p = 0.018, r = -.65) and the left amygdala ([-27 -1 26], p = 0.018, r = -.65 as well as a statistical trend in the right amygdala ([27 -4 -23] p = 0.078, r = -.52), which was not the case for the HCs (r = -.13, p>.5, z = 2.58, p<.05 for the insula and r = .11, p>.6, z = 1.88, p<.10 for the left amygdala; see Fig 4). With regards to the amygdala, BPD patients with low rejection sensitivity showed higher activation during pain after social exclusion compared with inclusion. However, as the rejection sensitivity increased, the activation became higher during pain after social inclusion compared with exclusion.

The regression analysis in the HC group did not reveal significant results in the pain-relevant ROIs.

## Discussion

The present study investigated whether the experiences of social inclusion and exclusion could modulate the subjective assessment of pain intensity and the neural processing of physical pain differently in BPD patients and HC. We observed a clear effect of social exclusion on subsequent pain processing in the form of increased pain sensitivity, which became apparent at a behavioral as well as on a neuronal level. The neural processing of physical pain after exclusion and inclusion was modulated in BPD patients by the individual level of rejection sensitivity.

In accordance with previous studies, we expected hyposensitivity to physical pain in BPD patients. In our study, both BPD patients and healthy controls received pain stimuli with a comparable subjective pain intensity of 60%. Consistent with the literature [12, 15, 17, 18, 44], BPD patients showed a higher pain threshold, i.e., higher temperature stimuli were applied to the BPD patients to induce an experience of pain that was comparable to that of healthy participants. On a neural level, BPD patients showed reduced activation in the amygdala, and increased activation of the right posterior insula during the processing of painful stimuli rather than non-painful stimuli. In accordance with our expectations, these effects were modulated by the nature of the preceding interaction situation; the increased insula activation during pain in BPD patients was linked to prior social exclusion, whereas reduced amygdala engagement during pain in BPD was most pronouncedly linked to prior social inclusion.

Overall, social exclusion was linked to a stronger self-reported experience of feeling excluded than social inclusion [26]. Because this effect was observed for both healthy subjects and patients with BPD, we expected the experience of social exclusion to affect pain processing in both groups in a more pronounced way than the experience of social inclusion or the participation in a rule-driven interaction situation. Our data reveal that social exclusion led to a subjective hypersensitivity to physical pain in both groups. This result is in line with the severity hypothesis by Bernstein and Claypool [8], which predicts a hyper- or hyposensitivity to pain depending on the strength of social exclusion. The authors classify the cyberball procedure as a mild form of social exclusion, which should therefore be linked to a hypersensitivity to physical pain. However, in contrast to our hypotheses, we found no group differences; i.e., both BPD patients and healthy subjects responded to the exclusion experience with a hypersensitivity to pain. This finding is consistent with the fact that exclusion ratings after social exclusion were comparable between groups, as has been described in a previous paper reporting on the social interaction findings of the present study [26]. This interpretation is also in line with a cyberball study by Lawrence et al. [45], who reported no difference between BPD patients and HC in terms of the intensity increase of negative emotions, and the intensity decrease of positive emotions after social exclusion.

Consistent with the behavioral data, our brain imaging data further support the severity hypothesis [8]. Independent of the group, we observed an enhanced activation in the anterior insula and the thalamus during the processing of physical pain after exclusion, in comparison with inclusion. The increasing activation in these regions was previously linked to increasing subjective pain intensity [46]. Whereas thalamus activation also relates to increasing stimulus intensity in PET studies [46], the anterior insula seems to play a crucial role in the affective pain component [47]. For both brain regions, Kross et al. [5] reported overlapping activation during social exclusion and physical pain. This finding is consistent with our observations of the increased engagement of these regions when experimentally inducing both physical and social pain. Taken together, social exclusion influences pain processing in brain regions linked to the affective as well as to the more sensory component of pain processing. This result suggests that social pain accentuates the experience of physical pain and the engagement of the related cerebral structures.

Beyond the modulation of physical pain processing in the anterior insula and the thalamus by the preceding social encounter in both BPD patients and HCs, both groups differ in the activation of some brain regions when comparing pain processing separately for the cyberball conditions.

Following social exclusion, brain responses to painful stimuli in the right posterior insula is stronger in BPD patients than in healthy participants. Posterior insula activation has been linked to the sensory-discriminative pain pathway, along with regions in the primary and secondary somatosensory cortex as well as the lateral thalamus [48]. The posterior insula has also been discussed as a brain region that is sensitive to the quality of painful stimuli, in contrast to non-painful stimuli. Specifically, this structure might serve to integrate information regarding the sensory intensity into the subjective experience of pain [49, 50]. Activation within this cerebral region has been shown to increase with pain intensity ratings of painful stimuli [50, 51]. In the present study, we could not replicate this finding. By contrast, in BPD subjects, insula activation increased with the temperature of the applied painful stimuli. The differential impact of the objectively applied temperature on insula activation in BPD patients and HC suggests that the integrative function of the posterior insula might be altered in BPD patients. Our data may thereby suggest that in BPD, the experience of social exclusion modulates the integration of sensory information into the subjective experience of pain via the posterior insula [49]. It is notable that we could not support our specific alteration of insula activation following exclusion in BPD patients when using three-fold interaction contrasts to compare group differences in pain processing after social exclusion to group differences in pain processing after social inclusion and after the control condition. Taken together with the significant insula effect on the group x temperature level, our inability to support the earlier hypothesis lends weight to the idea of a more general effect of social situations on the integrative function of the posterior insula in BPD. The fact that Schmahl et al. [12] did not observe activation differences in the insula during pain processing in BPD patients relative to HC reinforces the idea that this effect was caused in general by the precedence of social situations.

The experience of social exclusion has previously been shown to precede self-injurious behavior (SIB) in BPD patients [52] and is therefore directly connected to the experience of physical pain in the daily lives of these patients. BPD patients primarily engage in SIB to reduce inner tension or down-regulate negative emotions [53–55]. Niedtfeld et al. [56] proposed that this regulatory effect might be related to an attentional shift from the negative emotions towards the experience of physical pain. Although BPD is primarily associated with a hyposensitivity to physical pain, our data show that BPD patients can experience pain sensitization following social exclusion at least in the short term. This sensitization effect may be one reason why SIB is such a powerful emotion regulation strategy in BPD. Following the model of Ducasse et al. [57], this attentional shift is regulated through the insula. The enhanced insula activation during pain after exclusion in BPD might reflect this effect. These findings underline the potential for pain stimuli as regulatory skills in the form of non-invasive and non-harmful pain activation, which are already part of DBT skills training (e.g., pressuring pain points or using spiky massage balls to induce pain; [58]). These skills might be especially effective in regulating social pain because of the sensitization effect of social pain on the physical pain system.

Following social inclusion, BPD patients responded with a stronger activation decrease to painful stimuli than healthy subjects in the right amygdala. After social exclusion, this reduced amygdala activation was also evident; however, it was only observable as a statistical trend. As supported by a recent meta-analysis [39], the amygdala is consistently activated during experimentally induced pain. It has traditionally been linked to the affective pain component, but recent research suggests that the amygdala is also involved in more cognitive-evaluative pain processing, which may be related to its central position in the pain network. The amygdala receives input directly from the spinal cord and from the thalamus, insula, ACC, and prefrontal regions [39]. Although Schmahl et al. [12] reported reduced amygdala activation during pain in BPD patients, in our patient group, we only observed a trend for reduced amygdala activation during pain, which became statistically significant after social inclusion. These diverging results may be related to sample differences between both studies. Schmahl et al. [12] specifically included a sub-group of BPD patients who showed self-injurious behavior (SIB) and who reported a partial or complete absence of pain during SIB; only half the BPD sample fulfilled the DSM-IV criterion of SIB in the present study. Beyond that, far fewer subjects with a co-occurring posttraumatic stress disorder (approximately 50% of the sample in Schmahl et al. [12] and 15% in the current sample) may have contributed to inconsistent findings; Kraus et al. [59] observed significantly reduced amygdala activation during pain in BPD patients with co-morbid PTSD compared with BPD patients without co-morbid PTSD. These researchers proposed that the reduced amygdala activation during pain might be the relevant antinociceptive mechanism, particularly in BPD patients with co-morbid PTSD. Because they did not include a healthy control group, further studies are required to investigate the specificity of reduced amygdala activation during pain processing for BPD subjects with PTSD. The fact that we did find significantly reduced amygdala activation in BPD compared with HC during pain after social inclusion suggests that the experience of social situations that are unexpected for BPD patients, that is, situations of objective inclusion, led to an accentuation of already known alterations in the activation of the amygdala during pain [12]. Although BPD patients experienced a higher level of exclusion under this condition, alterations in amygdala activation cannot solely be linked to a higher level of reported exclusion, but they are related to the effects of the group, suggesting a different impact of social inclusion on pain processing in BPD patients. Our findings revealed differential effects during pain processing in the insula and the amygdala for both groups. However, our data did not confirm our hypotheses of group-specific activation differences in the thalamus, the ACC and the vl/dlPFC. Only the additional within-group analysis (see supporting information in S2 Text) provides first hints for possible group differences, but these effects could not be statistically supported in our between-group analysis and should therefore be interpreted with caution.

In the thalamus, which is associated with the sensory-discriminant pain component, we observed social exclusion effects on pain processing that were independent of BPD. This finding is consistent with previous studies suggesting that sensory-discriminant components of pain processing are not affected in BPD. It must be mentioned that the within-group analysis (see supporting information S2 Text) could support this effect only in the BPD group, and thus the group-independent effect of the primary analysis may be primarily triggered by the BPD group.

The activation of the ACC and the vl/dlPFC were neither differentially influenced by the group nor by the nature of the preceding social interaction. This result may suggest that social pain primarily influences the affective component of physical pain instead of processes such as threat detection and pain regulation and their related cerebral structures. One may speculate that missing group differences in the dlPFC are linked to the application of physical pain. Niedtfeld et al. [54] reported that physical pain normalized the coupling between (para-)limbic and prefrontal structures during the processing of negative emotional stimuli in BPD. The experience of physical pain may have enabled BPD patients to adapt the activation level of the dlPFC to that of the HC. Within-group comparisons of pain processing after exclusion and inclusion support this idea, revealing increased dlPFC activation in BPD patients during pain after social exclusion, which suggests a stronger engagement of the brain area linked to processes of cognitive control.

Finally, we were interested in finding whether rejection sensitivity modulates the effect of social exclusion and inclusion on physical pain processing. Given the link between the A118G polymorphism of the mu-opioid receptor gene, which was previously associated with both pain sensitivity and rejection sensitivity [11], we hypothesized that there might be a direct link between pain sensitivity and rejection sensitivity. Indeed, our findings suggest that rejection sensitivity is linked to a shift in the balance between the engagement of both the amygdala and the insula during pain after exclusion and inclusion. In BPD patients, increasing rejection sensitivity was associated with lower activation differences between pain after social exclusion and pain after social inclusion in the amygdala and the insula. Interestingly, these are the same regions we found to be differently activated during pain after different cyberball conditions between the BPD and the HC group. The observed lower activation differences imply that in BPD patients with low rejection sensitivity, activation is stronger during pain after social exclusion compared with inclusion. However, with increasing rejection sensitivity, the activation was observed to be stronger during pain after social inclusion than during pain after social exclusion.

One possible explanation for these findings may be derived from the severity hypothesis [8]. Accordingly, there is a shift in the influence of social exclusion on pain from sensitization to numbing, depending on the severity of exclusion; although experiences of mild exclusion lead to a higher sensitivity to pain, experiences of strong exclusion may decrease sensitivity. High rejection sensitivity implies that social exclusion is perceived more severely, possibly accounting for an earlier shift between sensitization and numbing in participants with high rejection sensitivity. The objectively identical exclusion condition may thereby result in both hypersensitization and hyposensitization, depending on the rejection sensitivity.

Our data confirmed the higher rejection sensitivity in BPD patients than in HC. Nevertheless, we observed a high variability in rejection sensitivity among BPD patients. One may speculate as to whether BPD patients with low rejection sensitivity react to the exclusion condition with a stronger engagement of the amygdala and the insula during pain compared with the inclusion condition because of the sensitization effect in response to the perception of a mild form of exclusion. With increasing rejection sensitivity, the same exclusion condition may be perceived as severe enough to shift sensitivity to numbness, as reflected in reduced amygdala and insula responses to pain after exclusion compared with inclusion.

Alternatively, one may speculate as to whether an increase in rejection sensitivity leads to a converging cerebral activation in response to social in- and exclusion, i.e., social inclusion and exclusion result in a similar cerebral hypersensitivity to pain. Our data suggest that when rejection sensitivity is high, the effects of inclusion on pain processing indeed exceed the effects of exclusion. One possible explanation may be that individuals who are highly sensitive to rejection expect to be rejected in social situations, and they may perceive inclusion as particularly unexpected and untrustworthy.

This interpretation is consistent with findings suggesting that in BPD, high rejection sensitivity is related to enhanced experiences of exclusion during situations of objective inclusion [13, 26]. Our data suggest that the RS most likely modulates both the processing of social exclusion and inclusion, and the subsequent processing of pain. However, these hypotheses are derived from our findings and remain to be confirmed. Of particular interest for future studies is a parametric variation in the level of exclusion through experimental manipulation, which would allow for the modeling of individual item response curves. Through those means, an analysis of the relations between rejection sensitivity and the intensity of exclusion may be achieved, including at which point the effect of exclusion on pain shifts from sensitization to numbing, which may provide data that add to the understanding of these processes.

Interestingly, rejection sensitivity primarily affected pain processing in the BPD patients, and not in the HC group. Because the HC had a considerably lower range of rejection sensitivity compared with the BPD group, it can be hypothesized that a certain level of rejection sensitivity is necessary to further influence pain processing, a level that may not be reached in our HC group.

Some limitations of the present study must be addressed. First, we applied a within-subject-design, i.e., all participants experienced phases of exclusion and inclusion. This finding might represent a less severe experience of exclusion compared with the results from MacDonald et al. [10]. Here, participants experienced either exclusion or inclusion, which may have increased the differential effect of exclusion and inclusion on pain. Additionally, we employed the same co-players during the whole experiment. This finding implies that the participants were uncertain about what behavior to expect next, with the highest uncertainty occurring during social inclusion (see also [26]). Although they reported feeling included during the inclusion blocks and excluded during the exclusion blocks, it cannot be ruled out that this uncertainty affected the processing of pain. Further studies should investigate whether the use of different teams of co-players results in a clearer discrimination between exclusion and inclusion, and a more pronounced differentiation of pain processing after social exclusion and inclusion.

Another limitation of this study is the lack of a control condition during which both groups experienced an equal level of inclusion. Because previous studies [13, 14] have already suggested that BPD patients may perceive inclusion conditions as less inclusive than HC, we added the control condition with predefined rules as an alternative comparison condition. Contrary to our expectations, BPD patients reported reduced experiences of inclusion compared with the HC, not only during the inclusion condition but also in this experimental control condition. Although this finding provided important evidence towards a rather generalized experience of exclusion in BPD, it hampers the evaluation of exclusion effects in this study because of the absence of a true inclusion comparison condition. However, it is important to note that both groups felt more excluded after the exclusion condition compared with the inclusion condition. This finding suggests that we were successful in manipulating the experience of social exclusion and inclusion in our study, although we found significant group differences in the level of experienced rejection after inclusion. Nevertheless, we cannot rule out the possibility that the different effects of social inclusion on pain in both groups might be related to the altered experience of inclusion and not to an altered effect of inclusion on pain processing. Further studies are required to investigate whether and under which conditions BPD patients may experience a comparable extent of being included such as healthy individuals to include an experimental condition that provides a more suitable control condition for feeling included.

Because both exclusion ratings and the RSQ-score strongly separate BPD patients and healthy subjects, the inclusion of exclusion ratings and RSQ scores as covariates in the analyses may result in an overcorrection, eliminating the between-group variance of interest linked to the BPD diagnosis.

Another limitation concerns the generalizability and specificity of our results. We only included female BPD patients, and thus it is not possible to generalize our findings to male BPD patients. Because we did not include a clinical control group, further studies must investigate whether our findings are specific for BPD. Several studies revealed alterations in the pain processing of people with depressive disorders (e.g., [60–62]. To avoid confounding the alterations of pain processing in BPD with those linked to depressive disorders, we excluded BPD subjects with a co-morbid major depressive episode from participating in the study at the cost of a reduced generalizability of our findings to BPD patients with concurrent depression.

Nevertheless, BPD is a complex disorder with heterogeneous symptomatology. Future studies must be focused on whether the processing of physical pain may be affected in a different manner by specific sub-groups of BPD patients. For example, self-injurious behavior, dissociation, childhood traumata, and a history of victimization in peer groups might differentially influence pain sensitivity following social exclusion [12, 15, 17, 44]. The sample size of the present study prevented an analysis of BPD subgroups depending on a history of self-harm with sufficient power. Nevertheless, exploratory analyses revealed a hyposensitivity to physical pain, particularly in BPD patients with past self-injurious behavior without any influence on the dependence of alterations in pain processing after different social encounters (see supporting information S1 Text). However, these exploratory analyses provide only hints towards the relevance of self-injurious behavior, and future studies must investigate the role of a history of self-harm in more detail. An animal model by Schneider et al. [63] has linked a history of social rejection during childhood and adolescence to a hyposensitivity to physical pain during adulthood. A comparable mechanism may match the development of alterations in pain processing in BPD because the experience of emotional neglect and social exclusion from the origin family or the early peer groups constitutes one of the central features in the biographies of BPD patients [64, 65].

In summary, this is the first study that has investigated the modulation of physical pain by social pain in BPD. Our data reveal an altered processing of pain in BPD patients, which is modulated by both the nature of the social context and rejection sensitivity, i.e., a cognitive affective predisposition characterizing BPD patients. Further research on BPD is needed to disentangle the specific mechanisms underlying the relations of physical and social pain processing and the influence of environmental factors and personality dispositions.

## Supporting Information

S1 Table t-contrast pain>warmth within the ROIs using a small volume correction (p_SVC-FWE_<.05). (DOCX)

S2 Table t-contrast pain>warmth, p<0.001, k>5, uncorrected. (DOCX)

S1 Text Additional sub-group analysis for BPD with and without self-injurious behavior. (DOCX)

S2 Text Additional within-group analysis of the fMRI data. (DOCX)
