# Supplementary material for: Pain Processing after Social Exclusion and Its Relation to Rejection Sensitivity in Borderline Personality Disorder
Source: PLoS One. 2015 Aug 4;10(8):e0133693. doi: 10.1371/journal.pone.0133693 (PMC4524681; doi:10.1371/journal.pone.0133693)
Supplement: S1 Table — (DOCX) [file pone.0133693.s001.docx]

**Supporting information**

**S1 Table: t-contrast pain>warmth within the ROIs using a small volume correction (p_SVC-FWE_<.05)**

|  |  |  |  | MNI | |  | |  | |
| --- | --- | --- | --- | --- | --- | --- | --- | --- | --- |
| BA |  | Anatomic Label | x | | y | | z | | T |
| ROI: ACC | | |  | |  | |  | |  |
| BA 24 | R | Anterior Cingulate | 0 | | 20 | | 28 | | 4.90 |
| BA 24 | L | Anterior Cingulate | -6 | | 29 | | 22 | | 4.67 |
| ROI: Insula |  |  |  | |  | |  | |  |
| BA 13 | R | Insula | 36 | | -19 | | 19 | | 11.28 |
| BA 13 | R | Insula | 39 | | -1 | | -8 | | 7.97 |
| BA 13 | R | Insula | 43 | | -1 | | 7 | | 7.35 |
| BA 13 | R | Insula | 36 | | 2 | | 10 | | 7.34 |
| BA 13 | R | Insula | 39 | | 14 | | -2 | | 6.82 |
| BA 13 | R | Insula | 45 | | 11 | | -2 | | 6.76 |
| * | L | Insula | -33 | | 17 | | 4 | | 6.29 |
| BA 13 | L | Insula | -36 | | -19 | | 16 | | 6.27 |
| BA 13 | L | Insula | -39 | | -4 | | 7 | | 6.12 |
| BA 13 | L | Insula | -36 | | -1 | | 10 | | 6.10 |
| BA 13 | L | Insula | -39 | | -1 | | -3 | | 5.82 |
| * | L | Claustrum | -33 | | 5 | | 10 | | 5.41 |
| BA 22 | L | Superior Temporal Gyrus | -48 | | -7 | | 4 | | 5.30 |
| BA 22 | L | Superior Temporal Gyrus | -48 | | 14 | | -2 | | 5.08 |
| BA 13 | L | Insula | -48 | | 8 | | 4 | | 5.07 |
| BA 47 | L | Inferior Frontal Gyrus | -36 | | 32 | | 7 | | 4.33 |
| ROI: Amygdala |  |  |  | |  | |  | |  |
| * | R | Amygdala | 24 | | -1 | | -14 | | 3.75 |
| * | R | Amygdala | 27 | | 2 | | -17 | | 3.65 |
| BA 34 | L | Parahippocampal Gyrus | -30 | | 2 | | -17 | | 3.02 |
| ROI: Thalamus |  |  |  | |  | |  | |  |
| * | R | Thalamus | 6 | | -10 | | 13 | | 4.87 |
| * | R | Thalamus | 9 | | -13 | | 4 | | 4.76 |
| * | R | Thalamus | 9 | | -23 | | 4 | | 4.14 |
| * | L | Thalamus | -6 | | -7 | | 10 | | 3.84 |
| * | L | Thalamus | -3 | | -13 | | 16 | | 3.83 |
| * | L | Thalamus | -9 | | -13 | | 4 | | 3.64 |
| * | L | Thalamus | -3 | | -25 | | 7 | | 3.59 |
| ROI: BA9 |  |  |  | |  | |  | |  |
| BA 9 | R | Middle Frontal Gyrus | 48 | | 8 | | 37 | | 5.08 |
| BA 45 | R | Inferior Frontal Gyrus | 57 | | 17 | | 23 | | 4.37 |
| BA 9 | L | Medial Frontal Gyrus | -6 | | 29 | | 37 | | 4.90 |
| BA 9 | L | Middle Frontal Gyrus | -42 | | 23 | | 34 | | 4.81 |
| BA 9 | L | Middle Frontal Gyrus | -48 | | 11 | | 31 | | 4.66 |
| BA 9 | L | Middle Frontal Gyrus | -45 | | 8 | | 37 | | 4.20 |
| BA 9 | L | Inferior Frontal Gyrus | -45 | | 2 | | 31 | | 4.04 |
| BA 32 | R | Cingulate Gyrus | 3 | | 29 | | 37 | | 4.80 |
