# Supplementary material for: Pain Processing after Social Exclusion and Its Relation to Rejection Sensitivity in Borderline Personality Disorder
Source: PLoS One. 2015 Aug 4;10(8):e0133693. doi: 10.1371/journal.pone.0133693 (PMC4524681; doi:10.1371/journal.pone.0133693)
Supplement: S2 Table — (DOCX) [file pone.0133693.s002.docx]

**Supporting information**

**S2 Table: t-contrast pain>warmth, p<0.001, k>5, uncorrected**

|  |  |  |  | MNI |  |  |
| --- | --- | --- | --- | --- | --- | --- |
| BA |  | Anatomic Label | x | y | z | T |
| BA 13 | R | Insula | 39 | -19 | 19 | 11.62 |
| BA 44 | R | Precentral Gyrus | 51 | -1 | 7 | 8.59 |
| BA 40 | R | Postcentral Gyrus | 60 | -19 | 19 | 8.50 |
| * | R | Cerebellum | 33 | -70 | -41 | 5.80 |
| * | L | Cerebellum | -27 | -67 | -29 | 5.32 |
| * | L | Cerebellum | -39 | -73 | -29 | 5.12 |
| BA 5 | R | Superior Parietal Lobule | 21 | -43 | 67 | 4.30 |
| BA 22 | R | Middle Temporal Gyrus | 60 | -40 | 1 | 4.05 |
| BA 41 | R | Superior Temporal Gyrus | 45 | -43 | 7 | 3.57 |
| BA 21 | L | Middle Temporal Gyrus | -63 | -34 | -5 | 4.01 |
| BA 21 | L | Middle Temporal Gyrus | -57 | -40 | -2 | 4.00 |
| BA 7 | R | Precuneus | 12 | -64 | 40 | 3.98 |
| BA 17 | R | Lingual Gyrus | 12 | -94 | -8 | 3.83 |
| BA 7 | L | Precuneus | -9 | -67 | 40 | 3.74 |
