# Supplementary material for: Pain Processing after Social Exclusion and Its Relation to Rejection Sensitivity in Borderline Personality Disorder
Source: PLoS One. 2015 Aug 4;10(8):e0133693. doi: 10.1371/journal.pone.0133693 (PMC4524681; doi:10.1371/journal.pone.0133693)
Supplement: S1 Text — (DOCX) [file pone.0133693.s003.docx]

**Supporting information**

**S1 Text. Additional sub-group analysis for BPD with and without self-injurious behavior**

For explorative purposes, we conducted subgroup-analysis of our behavioral data, dividing the BPD group into patients who fulfilled the DSM-IV criterion of self-harm behavior and those who did not. The groups for these analysis consisted of n=10 BPD patients with self-harm behavior (BPD+), n=10 BPD patients without self-harm behavior (BPD-) and n=20 healthy control subjects (HC).

To compare pain temperatures corresponding to 60% pain intensity between groups, a one-way-ANVOA was conducted and revealed a significant effect for group (F(2,37)=4.44; p=.019). The highest mean temperature was seen in the BPD+ group (M=44.75, SD=1.8), followed by the BPD- group (M=43.80, SD=1.8) and the HC group (M=42.50, SD=2.0). Bonferroni-corrected post-hoc tests show significant higher temperatures in the BPD+ group compared to HC (p=.020). Group differences between BPD+ and BPD- and between BPD- and HC did not reach significance (p=.896 and p=.313).

Differences in the subjective pain ratings of the painful stimuli after the cyberball conditions were tested using 3x3-repeated-measures analyses of variance with the between-subject factor ‘group’ (BPD+, BPD-, HC) and the within-subject factor ‘cyberball condition’ (exclusion, inclusion, control). According to our main analysis, subjective pain ratings were only modulated by the preceding interaction situation (F(2,74)=8.75, p<.001). There was no significant group (F(2,37)=1.34, p=.274) or group x condition interaction effect (F(4,74)=.281, p=.890).
