# Supplementary material for: Pain Processing after Social Exclusion and Its Relation to Rejection Sensitivity in Borderline Personality Disorder
Source: PLoS One. 2015 Aug 4;10(8):e0133693. doi: 10.1371/journal.pone.0133693 (PMC4524681; doi:10.1371/journal.pone.0133693)
Supplement: S2 Text — (DOCX) [file pone.0133693.s004.docx]

**Supporting information**

**S2 Text. Additional within-group analysis of the fMRI data**

Due to the reduced comparability of the inclusion condition as control that provides similar experiences of being included between groups, we conducted additional within-group analyses to compare pain after social exclusion with pain after social inclusion and after the control condition. Additional analyses contrasting experimental conditions separately for each group supported our findings of the condition x temperature interaction of the main analysis for the BPD group. The BPD patients showed enhanced activation during pain (vs. warmth) after social exclusion compared to pain (vs. warmth) after social inclusion in the left anterior insula ([-30 23 -2], p_SVC_=.010) and the right thalamus ([9 -10 16], p_SVC_=.039). Similarly, we observed enhanced activation in the left dlPFC (BA9, [-30 23 -2], p_SVC_=.010) and vlPFC (BA47, [-30 20 -2], p_SVC_=.015). Contrarily in the HC group, neural activation was not distinguishable between pain (vs. warmth) after exclusion and after inclusion (all p_SVC_>.05). Comparing pain (vs. warmth) after exclusion with pain (vs. warmth) after the control condition revealed only in the BPD group a significant effect in the right ([27 2 -20], p_SVC_-FWE=.005) and left amygdala ([-21 -1 -17], p_SVC_-FWE=.020), which can be explained by a reduced amygdala activation during warmth after the control condition, comparable with the same contrast in the main analysis of the whole sample (interaction effect condition x temperature). Additionally, BPD patients showed enhanced activation during pain (vs. warmth) after exclusion compared to the control condition in the right insula ([42 -40 19], p_SVC_=.031) and the right vlPFC (BA47, [-30 38 -11], p_SVC_=.001).Again, in the HC group, neural activation was not distinguishable between pain (vs. warmth) after exclusion and after the control condition (all p_SVC_>.05).
